# Tapestry of postnatal emotional disorders: exploring the interplay of anxiety and depressive disorders and their associated risk factors in Sudanese women

**DOI:** 10.3389/fpubh.2024.1446494

**Published:** 2024-09-24

**Authors:** Abdelgadir H. Osman, Abdelaziz Osman, Ibtihal A. Osman, Taisir Hagar

**Affiliations:** ^1^Department of Psychiatry, Faculty of Medicine, University of Khartoum, Khartoum, Sudan; ^2^Al Amal Psychiatric Hospital, Dubai, United Arab Emirates; ^3^Faculty of Medicine, Gezira University, Wad Madani, Sudan

**Keywords:** postnatal depression, postnatal anxiety, postnatal emotional disorders, Sudan metal health, women mental health, post-partum anxiety and depressive disorder, mixed anxiety and depressive disorder

## Abstract

**Background:**

This research aims to unravel the prevalence of postnatal emotional disorders with a focus on how postnatal anxiety remained under-estimated and often embroiled in postnatal depression.

**Methods:**

Out of 600 postnatal women invited to take part in this study from two prominent primary care clinics in Khartoum, 468 women agreed to participate in this study. Three questionnaires were utilized in this study, a Personal Information Questionnaire (PIQ), Hospital Anxiety and Depression Scale (HADS), and Beck depression Inventory (BDI). Multiple linear regression analysis applied to gauge risk factors with postnatal anxiety and depression.

**Results:**

More than half (52.50%) of women showed evidence of both anxiety and depression using HADS, while only (20.9%) of cases were detected by BDI, showing evidence of moderate depressive disorder. A substantial proportion (28.4%) showed high levels of comorbidity of anxiety and depression in the category of moderate to severe symptoms. Main risks factors for postnatal disorders were past psychiatric illness (β = 0.25, *p* = 0.001), a family history of psychiatric illness (β = 0.15, *p* = 0.002), and stress due to the number of children (β = 0.32, *p* = 0.001).

**Conclusion:**

This study advances our understanding of postnatal emotional disorders, particularly highlighting the prevalence as well as correlates of postpartum anxiety. More importantly, this study highlights the importance of routine screen for emotional distress in postnatal women.

## 1 Introduction

In the realm of maternal mental health, the postnatal period stands as a unique and critical juncture marked by multifaceted challenges and emotional complexities (1–3). According to existing literature, ~10%−20% of women are expected to experience distressing emotional disorders in the postnatal period (4). The severity of such disorders could be gauged from the fact that in the United Kingdom, 9% of postnatal maternal mortality is attributed to mental health disorders (5).

The existing literature predominantly centers on the occurrence, predictors, and repercussions of perinatal mood states, with a primary focus on postpartum depression (6–8). As the body of research is evolving, there has been a growing recognition of the importance of exploring additional prevalent mood states, such as anxiety and comorbid anxiety and depression (9, 10). A recent meta-analysis sponsored by the Royal College of Psychiatry found an average prevalence of 15.1% of postnatal anxiety symptoms and 9.1% of generalized anxiety disorders (11). The significance of conducting further research on maternal anxiety during the postnatal period was underscored by (12), who identified a predictive relationship between elevated anxiety levels and low birth weight (12). Moreover, many researchers demonstrated time and again that postnatal anxiety is tagged with an increased risk of suicide, as well as reduced mother-infant interactions, bonding problems, abnormal infant temperament, mother's mental, and child health and future development (13). It has been confirmed by experimental psychological research how anxiety disorder in postnatal mothers affects mother-child relationships leading to less communication, disrupting care and with a negative impact on the emotional development of children (14). This effect was believed to be mediated by negative worrying cognition (14). On the other hand, it has been shown that mothers with postnatal anxiety show maladaptive and inappropriate coping mechanisms leading to dysfunctional relationships with their developing children (15). At the same time, postnatal depression has also been linked to adverse outcomes such as low infant body weight, and increased physical morbidity in infants, including conditions like diarrhea and vitamin deficiencies (16).

Moreover, epidemiological research consistently reveals elevated levels of co-occurrence between depressive and anxiety disorders (17–19). A study in the Netherlands found that among individuals having a depressive disorder, a whopping 67% concurrently experienced an anxiety disorder (20). Conversely, among those who have an anxiety disorder as well, 63% also had a concurrent depressive disorder (21). Moreover, the study highlighted that in 57% of cases with co-existing conditions, anxiety manifested before depression, while in 18%, depression came before anxiety. Findings from the U.S.-based National Comorbidity Survey, involving over 8,000 individuals in community settings, support the notion that anxiety disorders usually come before depressive disorders (22). Despite the escalating prevalence of postpartum anxiety and the documented co-occurrence of postnatal anxiety with postnatal depression, both of which have adverse impacts on child development, there remains a lack of comprehensive research on these issues particularly in the context of low- and middle-income countries (23).

The present study stands as the first study of post-natal anxiety as well as the comorbidity of postnatal anxiety and depression in Sudan to better understand its clinical correlates. The objectives of the research are to understand the prevalence of postpartum anxiety disorder independently from postnatal depression and estimate the prevalence of the comorbid state with depression.

## 2 Methods

### 2.1 Subjects and instruments

The present study involved the recruitment of participants from two prominent postnatal clinics situated in the capital city of Sudan, Khartoum. Potential participants were approached and provided with a detailed leaflet elucidating the study's objectives, the nature of the questionnaires employed, and the assurance of strict confidentiality. Out of the 600 women who visited these clinics during the four-month study period (April–August 2014), 468 voluntarily agreed to participate in the study. Therefore, all postnatal women who visited these centers during the study period were invited to take part in the study. Only those, who declined to take part on the study were exclude.

Upon consenting to partake in the research, women were asked to complete a comprehensive Personal Information Questionnaire (PIQ), designed to gather sociodemographic details, information regarding past and present psychiatric illnesses, family medical history, existing social support structures, and current financial status. The assessment of anxiety and depression levels was conducted using the HADS, which comprises two subtests specifically targeting anxiety (A) and depression (D). Additionally, the intensity of depressive symptoms was independently measured using the BDI. HADS scale has undergone validation as a highly sensitive tool for identifying symptoms related to depression and anxiety, contrasting using the Edinburgh Depression Scale (EDS), which primarily demonstrates sensitivity specifically to depressive disorders during the perinatal period. Additionally, HADS as well as BDI have been validated within the cultural context. Therefore, our methodology is aimed to ensure a rigorous and thorough examination of the participants' postnatal emotional well-being, considering various socio-demographic factors and psychiatric history (16, 24, 25).

### 2.2 Assessments

Assessments were carried out by two psychiatrists assisted by 4 research psychologists. Three-day training course and pilot study resulted in a high inter-rater reliability of 96% between the research team and data collectors. During the study, participants engaged in one-on-one sessions to fulfill the requirements of the research, which included the completion of the PIQ, HADS, and BDI. Throughout these interactions, any queries posed by the participants were met with sensitive responses, and pertinent guidance was provided as needed. The PIQ was employed to collect comprehensive socio-demographic data concerning candidates, with a specific focus on significant personal, familial, and current psychiatric histories, as well as physical ailments, in addition to social stressors and situations.

The second survey utilized standardized HADS to assess symptoms of anxiety and depression, gauging distress levels through self-reported responses. This instrument is psychometrically robust in identifying emotional distress during the postnatal stage. Comprising fourteen items rated on a Likert-type scale with 4 points, HADS includes subscales for anxiety and depression assessment. The seven items for each subscale result in a score ranging from 0 to 21, with designated cut points: normal (0–7), mild mood disturbance (8–10), moderate mood disturbance (11–14), and severe ones (12–21). The HADS scale is widely used as a sensitive tool for detecting anxiety and depression symptoms, particularly in comparison to the widely used Edinburgh Depression Scale (EDS), which primarily focuses on depressive symptoms in the peripartum. HADS as well as BDI instruments have undergone successful validation within the Sudanese cultural context.

In addition, a third questionnaire, BDI, was employed to confirm/validate the depressive symptoms reported and identified by HADS and to understand the extent of depressive illness. BDI, consisting of 21 items containing a Likert-type scale, records symptoms on a scale from 0 to 3 (0 indicating the absence of symptoms and 3 signifying severe symptoms). BDI yields a score from 0 to 63, and a score (18–23) indicates moderate depression, while a score (23) and above indicates severe depression.

### 2.3 Compliance with ethical standards

All the performed procedures contained in this study were according to the ethical standards of the Research Board of the Faculty of Medicine, University of Khartoum, and that of the Helsinki Declaration 1964, along with its later amendments or comparable ethical standards. Formal permissions were received from all relevant hospital administrations. Written consent was taken from all participants, who were provided with an information leaflet explaining the study procedures and confidentiality.

### 2.4 Data analysis

Analysis of data was performed using SPSS Version 22.0 to get quantitative and descriptive statistical measurements. Student *t*-test and Chi-square test were measured along *p*-values for significant correlates. The percentage of prevalence was worked out besides calculating frequencies of important correlates and indicated significance via *p*-values. Furthermore, Multiple linear regression analysis was applied to gauge risk factors for postnatal anxiety and depression. This was applied to most well cited risks factors associated with these disorders. The prevalence of anxiety was determined through the HADS A subtest, and the deduction of moderate to severe values of BDI cases from the total patients with high comorbid values in HADS total scores further contributed to the comprehensive analysis of anxiety prevalence. Further, regression tests were carried out to gauge potential risk factors and causal associations.

## 3 Results

### 3.1 Social demographic characteristics

**Figure 1:**
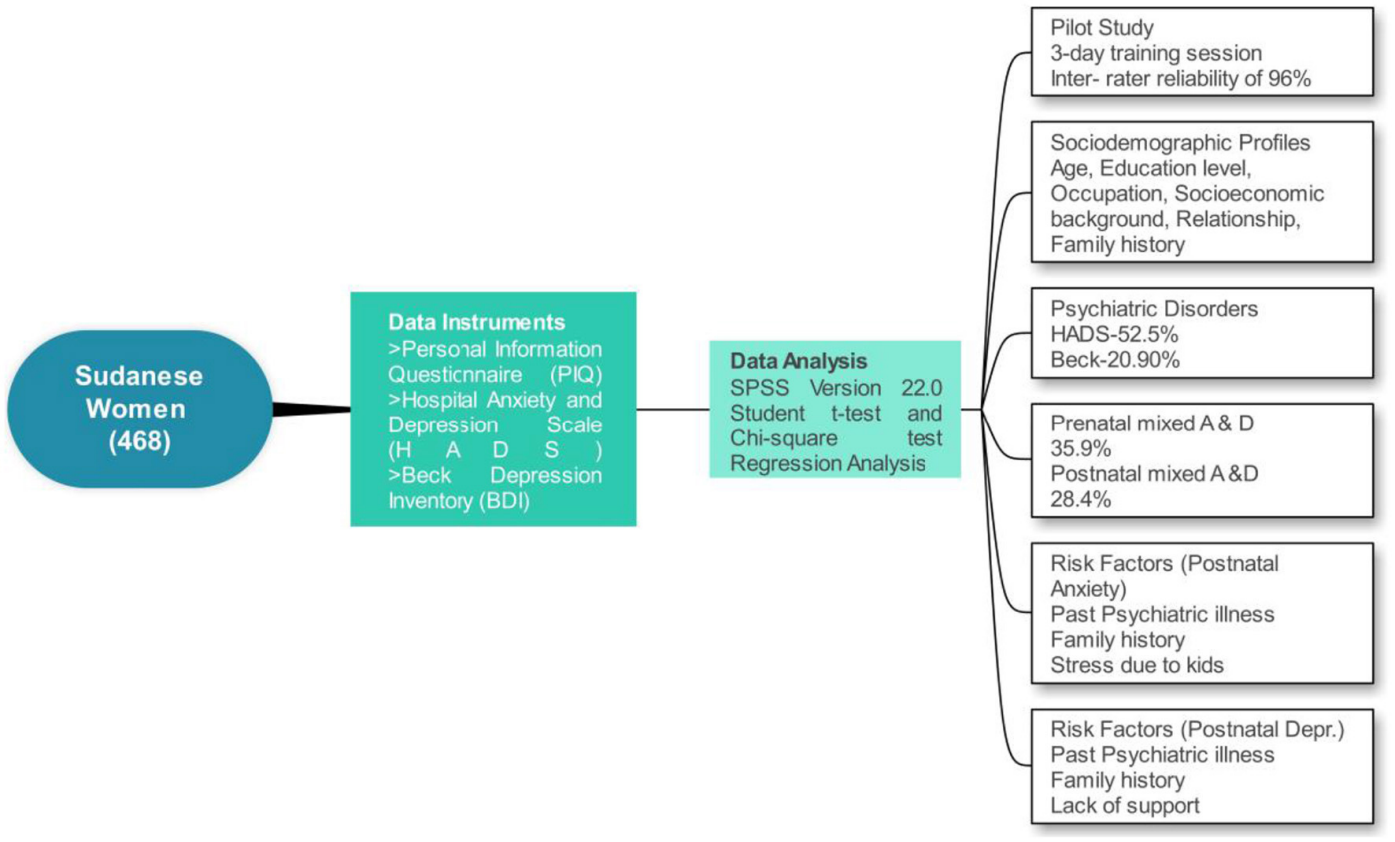
Sketch of methods and results of the study.

Table 1 presents a comprehensive overview of the social demographic characteristics of the study participants. Most women in the study fell within the 25–35 age range, comprising 59.14% of the sample (mean age 36.4 ± 1.6). In terms of education, a significant proportion of participants were undergraduates (88.17%), while 11.83% reported being illiterate. This diversity in educational backgrounds is crucial for understanding potential variations in health literacy and coping mechanisms (Figure 1).

**Table 1:** Socio-demographic characteristics.

**Characteristic**	**Number**	**Percentage (%)**
**Age**		
25–35 years	275	59.14%
36–45 years	142	30.54%
46–55 years	48	10.32%
**Educational level**		
Undergraduates	410	88.17%
Illiterates	55	11.83%
**Occupation**		
Housewives	225	48.39%
Other	240	51.61%
**Socioeconomic background**		
Low	327	70.32%
Medium	89	19.14%
High	49	10.54%
**Presence of a helping hand at home**		
Yes	217	46.67%
No	248	53.33%
**Relationship with husband**		
Good	371	79.78%
Average	64	13.79%
Poor	30	6.43%
**Family history of psychiatric disorders**		
Yes	282	60.65%
No	183	39.35%

### 3.2 Clinical characteristics

Table 2 provides insights into the distribution of psychiatric disorders (either anxiety/depression or comorbid condition), revealing that more than half of the cases (52.50%) are detected using HADS while 221 (47.5%) did not show high symptoms of depression or anxiety. This underscores the significance of employing a comprehensive assessment tool like HADS, which captures a broader spectrum of emotional distress, including anxiety and depression. Beck, while contributing significantly, reflects a narrower focus (20.9%) on depressive disorders.

**Table 2:** Distribution of psychiatric disorders.

	**Number**	**Percentage**
HADS	244	52.50%
Beck	97	20.90%
Total	465	100.00%

Table 3 provides a comprehensive overview of the prevalence of mixed depression and anxiety symptoms during postnatal periods, employing the HADS with the severity levels encompassing normal, mild, moderate, and severe symptoms. According to the results, 28.4% showed high levels of distress postnatally showing moderate to severe symptoms.

**Table 3:** Mixed anxiety and depressive symptoms' prevalence in postnatal period using HADS.

**Severity level**	**Postnatal (number)**	**Postnatal (percentage)**
Normal	261	56.13%
Mild	132	28.39%
Moderate	84	18.06%
Severe	48	10.32%
Total	465	100.00%

Table 4 presents a correlation analysis of various risk factors with postnatal anxiety and depression among study participants. The findings reveal several noteworthy associations. Participants with a history of psychiatric illness exhibit a strong positive correlation with postnatal anxiety (HADS) and a moderate correlation with depression (Beck), suggesting a connection between past mental health experiences and current emotional wellbeing. Similarly, a family history of psychiatric illness demonstrates a positive correlation with postnatal anxiety and depression, again with a slightly stronger association observed for anxiety.

**Table 4:** Correlation analysis of risk factors with postnatal anxiety and depression.

**Risk factors**	**Postnatal anxiety (HADS)**	**Postnatal depression (Beck)**
Past psychiatric illness	0.45	0.25
Family history of psych. illness	0.45	0.35
Lack of support	0.35	0.35
Hostile non-supportive partner	0.43	0.32
Low socioeconomic status	0.44	0.25
Stress due to the number of kids	0.55	0.30
Physical morbidity	0.32	0.24

In Table 5, the regression coefficients (β) reveal significant insights into the factors influencing postnatal anxiety. Notably, a history of past psychiatric illness (β = 0.25, *p* = 0.000), a family history of psychiatric illness (β = 0.15, *p* = 0.002), and stress due to the number of children (β = 0.32, *p* = 0.000) emerge as significant positive predictors of postnatal anxiety. These findings suggest that addressing historical psychiatric issues, family history, support systems, and socioeconomic factors is crucial in managing postnatal anxiety.

**Table 5:** Regression analysis pertaining to risk factors for postnatal anxiety (HADS).

	**Regression coefficients (β)**	***p*-value**
Past psychiatric illness	0.25	0.000
Family history of psych. illness	0.15	0.002
Lack of support	−0.15	0.035
Hostile non-supportive partner	−0.12	0.020
Low socioeconomic status	−0.25	0.002
Stress due to number of kids	0.32	0.000
Physical morbidity	0.25	0.055

Table 6 focuses on postnatal depression, with results indicating distinct predictor impacts. Past psychiatric illness (β = 0.52, *p* = 0.000), family history of psychiatric illness (β = 0.25, *p* = 0.02), and stress due to the number of children (β = 0.36, *p* = 0.002) are identified as significant positive contributors to postnatal depression. Lack of support (β = 0.17, *p* = 0.005) and a hostile non-supportive partner (β = 0.15, *p* = 0.002) also exhibit significant positive associations. In contrast, low socioeconomic status (β = 0.028, *p* = 0.008) demonstrates a weaker positive association and physical morbidity shows no significant impact on postnatal depression.

**Table 6:** Regression analysis pertaining to risk factors for postnatal depression (Beck).

	**Regression coefficients (β)**	***p*-value**
Past psychiatric illness	0.52	0.000
Family history of psych. illness	0.25	0.02
Lack of support	0.17	0.005
Hostile non-supportive partner	0.15	0.002
Low socioeconomic status	0.028	0.008
Stress due to number of kids	0.36	0.002
Physical morbidity	0.12	0.095

Table 7 provides an understanding of the distribution of risk factors and the prevalence of mixed anxiety and depressive symptoms during the postnatal period. Notably, the number of children in a family exhibits a gradient effect on symptom severity, with 52 cases classified as normal, 75 as mild, 85 as moderate, and 20 as severe in families with 0–4 children. In families with 5–10 children, 34 cases are normal, 17 are mild, 21 are moderate, and 28 are severe. This highlights a progressive increase in symptom severity with larger family sizes. The quality of the marital relationship emerges as a pivotal factor, with the absence of normal cases in poor relationships and an escalation of severity from mild to severe.

**Table 7:** Risk factors associated with mixed anxiety and depression.

**Risk factors**	**No. of children**		**Marital relation-ship**			**Past psychiatric depression**		**Co-morbidity with pregnancy**		**Family history of psychiatric illness**		**Physical illness**	
	**0–4**	**5–10**	**Good**	**Average**	**Poor**	**Yes**	**No**	**Yes**	**No**	**Yes**	**No**	**Yes**	**No**
Normal	52	34	62	14	0	13	80	13	80	23	80	116	349
Mild	75	17	76	4	0	23	98	80		21	115	–	–
Moderate	85	21	142	18	0	37	83	24	97	38	55	–	–
Severe	20	28	100	45	4	42	89	31	140	48	85	–	–
Total	232	100	380	81	4	115	350	148	317	130	335	116	349

Individuals with a history of psychiatric depression face heightened vulnerability, evident in the higher prevalence of severe symptoms (42 cases) compared to those without such a history (89 cases). Co-morbidities with pregnancy amplify symptom severity, emphasizing the need for integrated healthcare approaches. Family history of psychiatric illness consistently influences severity levels, with individuals having a family history displaying higher prevalence across various severity levels.

## 4 Discussion

This study represents the first comprehensive investigation into postnatal anxiety and depression among Sudanese women, addressing a significant research gap in the understanding of maternal mental health within this population in under-resourced settings. Through a meticulous examination of sociodemographic factors and psychiatric history, we aimed to understand the prevalence, correlates, and comorbidity of postpartum emotional disorders, particularly focusing on anxiety and depression. Employing a robust methodology, we recruited participants from prominent postnatal clinics in Khartoum and administered validated assessment tools, including the HADS and the BDS, to measure anxiety and depression symptoms.

The distribution of psychiatric disorders (either depression/anxiety or mixed condition) showed 52.50% of the cases as detected using HADS while Beck reflected a narrower focus (20.9%) on depressive disorders. In the postnatal period, a substantial proportion (28.4%), showed high levels of distress in the category of moderate to severe symptoms. Family history of psychiatric illness and past psychiatric history of disorders correlated positively with the emergence of emotional disorders. The results revealed that apart from depression as a widely acknowledged mental health issue in the postnatal period, the manifestation of anxiety alone or in the co-morbid state with depression is significant. These findings underscore the importance of tailored interventions specifically for the vulnerable population in resource-limited countries. Therefore, in addition to focusing on postnatal depression which has been studied more frequently in the postnatal context, we must recognize and address postnatal anxiety and comorbid states, given their high prevalence (as observed in our study) and impact on maternal mental health. Our recent study underscores the importance of considering comorbid conditions, particularly in resource-limited countries.

Postnatal anxiety disorders, as revealed in our research, constitute a significant portion of postnatal emotional disorders and merit equal attention in maternal mental health initiatives. In a review of pre- and postnatal psychological wellbeing in Africa, depression was found to be the most studied disorder, with prevalence rates of 18.3% after birth and 11.3% before birth (pregnancy). Rates for anxiety in the prenatal and postnatal periods were 14.8% and 14.0%. Higher prevalence was observed in low- to middle-income countries (11) which is also corroborated by this study.

Furthermore, the co-existence of postpartum anxiety and depression was identified in various studies. For instance, in a study involving 798 women, a significant association was identified between postnatal anxiety and depression calculated according to DSM-IV criteria (26). Moreover, in women from a maternity ward, 40% of postpartum individuals exhibited elevated anxiety which was diagnosed by the State-Trait Anxiety Inventory, and a noteworthy correlation was found between postnatal anxiety and depression (27). In a more extensive survey, 18% of 4,451 postnatal women showed symptoms of postnatal anxiety (28). Among these, 35% also reported symptoms of postpartum depression. This work highlights the significance of some correlated factors in relation to the emergence of postnatal emotional distress that were previously found in different cultures. Moreover, it sheds light on the understanding of the comorbid nature and spectrum of symptoms that this condition presents with.

The literature extensively investigates various risk factors associated with postpartum depression. Strong predictors include a history of depression or anxiety during pregnancy, as well as a previous depressive illness. Additionally, life stress and inadequate social support demonstrate a moderate to severe effect size in predicting postpartum depression (29, 30). In this study, hostile and non-supportive partners, family history and past psychiatric history of affective disorder correlated positively with the emergence of post-natal emotional disorders in agreement with previous findings, none of the other sociodemographic factors correlated significantly with the occurrence of postnatal emotional disorder.

The findings of this study contribute valuable insights into the prevalence and correlates of postnatal emotional disorders, particularly focusing on anxiety. The investigation addressed a critical gap in existing research, particularly in the context of developing countries, with a specific focus on Sudan. Additionally, the study shed light on the coexistence of anxiety and depression, emphasizing the need to explore these emotional disorders as a spectrum rather than isolated conditions.

The comorbidity of anxiety and depression during the postnatal period aligns with global research trends, with over 67% of comorbidity reported in community studies in the United States. One can fail to detect the extent of the emotional disturbance due to the use of an instrument that selectively detects one type of emotional disturbance either depression or anxiety. On the other hand, most postnatal instruments were found to be non-highly specific in detecting what supposed to measure.

Another important aspect to consider is the recent studies challenging the conventional nomothetic perspective on postnatal emotional disorders, positing that these conditions are not exclusively depressive or anxiety disorders. Instead, they are better comprehended as a spectrum of emotional disorders characterized by a blend of depressive and anxiety symptoms, although occasionally manifesting in distinct forms. This nuanced conceptualization sheds light on the heterogeneous nature of postnatal emotional disorders, offering a more accurate and comprehensive framework for understanding the diverse manifestations of these conditions (31, 32). Future research on postnatal emotional disorders particularly in low-income countries needs to consider such reports.

## 5 Limitations of the study

The study's limitations warrant consideration. Firstly, the sample size, drawn from two primary care clinics in Khartoum, Sudan, may limit the generalizability of findings to broader populations of postnatal women, possibly overlooking diverse experiences and backgrounds. Moreover, reliance on self-reported measures for assessing anxiety and depression levels introduces potential biases, as well as limitations in reliance on self-report measures namely the HADS and the BDI, which operate on a self-report basis and may not always accurately represent the actual clinical diagnosis of postnatal emotional disorders. Addressing these limitations in future research could enhance understanding and intervention strategies for postnatal emotional disorders among diverse populations of women.

## 6 Conclusion

The distribution of psychiatric disorders (either depression/anxiety or mixed condition) showed that more than half of the cases (52.50%) were detected using HADS while 221 (47.5%) did not show significant symptoms clinically. Beck, while contributing significantly, reflects a narrower focus (20.9%) on depressive disorders. In the postnatal period, a substantial proportion (28.4%), showed high levels of distress in the category of moderate to severe symptoms. Family history of psychiatric illness and past psychiatric history of disorders correlated positively with the emergence of emotional disorders. In agreement with previous findings, none of the other sociodemographic factors correlated significantly with the occurrence of postnatal emotional disorder.

## Data Availability

The original contributions presented in the study are included in the article/supplementary material, further inquiries can be directed to the corresponding author.
